# The effects of creatine supplementation with and without an Extract of *Artemisia dracunculus* on resistance training adaptations: preliminary findings

**DOI:** 10.1186/1550-2783-10-S1-P21

**Published:** 2013-12-06

**Authors:** Adam G Parker, Taylor Steele, Ralf Jäger, Martin Purpura, Allyn G Byars

**Affiliations:** 1 Department of Kinesiology, Angelo State University, San Angelo, TX 76909, USA; 2 Increnovo LLC, 2138 E Lafayette Pl, Milwaukee, WI 53202, USA

## Background

Co-ingesting creatine (5 g) with large amounts of glucose (e.g., 95 g) has been shown to enhance creatine and carbohydrate storage in muscle. It has been speculated that creatine transport is mediated in part by glucose and insulin. The increases in creatine retention are accompanied by an undesired caloric load and as a result, additional research has been undertaken to assess the effect of co-ingesting creatine with nutrients that may enhance insulin sensitivity. Co-ingestion of creatine (Cr) with an antihyperglycemic extract of Artemisia dracunculus (Russian tarragon (RT)), has been shown to influence plasma Cr levels comparable to co-ingestion of Cr and glucose [1]. However, other research has shown that short term (5 days) co-ingestion of Cr and RT (Cr+RT) did not enhance whole body creatine retention or muscle free creatine content [2]. The purpose of this on-going investigation was to compare the long-term effects of resistance training in combination with either Cr+RT, or Cr with carbohydrate (Cr+CHO), or carbohydrate (PL) ingestion.

## Methods

In a randomized, double-blind manner, 12 resistance trained males (n=8) and females (n=4) consumed either 90 g/day of dextrose + 0.38 g/day of fruit punch flavoring (PL, n=5), 84 g/day of dextrose + 6 g/day of Cr + 0.38 g/day of fruit punch flavoring (Cr+CHO, n=4), or 1,100 mg/day of RT + 6 g/day of Cr + 40 g/day of hydrolyzed collagen + 0.38 g/day of fruit punch flavoring (Cr+RT, n=3) for 8 weeks. Participants performed 4 days per week (2 upper-body, 2 lower-body) of resistance training. Body composition via DEXA, 1 repetition maximum (1RM) on bench press and back squat, and anaerobic power were measured at weeks 0, 4, and 8. Delta scores for all dependent variables were analyzed using individual ANOVAs.

## Results

Increases in lean body mass were significantly higher (p=0.038) after 4 weeks in the Cr+CHO (1.56 + 0.64 kg) and the Cr+RT (1.87 + 0.98 kg) groups compared to PL (0.02 + 1.08 kg). There were no other significant effects due to supplementation on body composition, 1RM bench press, 1RM back squat, or anaerobic power. Additionally, the Cr+RT group showed average improvements in strength to be equal to or greater than Cr+CHO. Also, by the end of the study, body fat decreased in the Cr+RT group (-2.42 + 6.81 kg), while the other two groups showed increases in body fat (Cr+CHO 0.83 + 0.79 kg, PL 1.10 + 0.88 kg), potentially linked to the increased caloric load.

## Conclusion

Although there was a limited sample size for each supplement group, preliminary data suggests that consuming Cr+RT is as effective as consuming Cr+CHO in regards to gains in LBM and strength over the course of 8 weeks of resistance training.
